# Pediatric high-grade gliomas and the WHO CNS Tumor Classification—Perspectives of pediatric neuro-oncologists and neuropathologists in light of recent updates

**DOI:** 10.1093/noajnl/vdac077

**Published:** 2022-05-20

**Authors:** Gerrit H Gielen, Joshua N Baugh, Dannis G van Vuurden, Sophie E M Veldhuijzen van Zanten, Darren Hargrave, Maura Massimino, Veronica Biassoni, Andres Morales la Madrid, Michael Karremann, Maria Wiese, Ulrich Thomale, Geert O Janssens, André O von Bueren, Thomas Perwein, Gunther Nussbaumer, Eelco W Hoving, Pitt Niehusmann, Marco Gessi, Robert Kwiecien, Simon Bailey, Torsten Pietsch, Felipe Andreiuolo, Christof M Kramm

**Affiliations:** Institute of Neuropathology, Medical Center Bonn, Bonn, Germany; Princess Máxima Center for Pediatric Oncology, Utrecht, The Netherlands; Princess Máxima Center for Pediatric Oncology, Utrecht, The Netherlands; Princess Máxima Center for Pediatric Oncology, Utrecht, The Netherlands; Erasmus University Medical Center, Department of Radiology and Nuclear Medicine, Rotterdam, The Netherlands; Great Ormond Street Hospital for Children, NHS Trust, London, UK; Fondazione Istituto di Ricovero e Cura a Carattere Scientifico, Istituto Nazionale dei Tumori, Milan, Italy; Fondazione Istituto di Ricovero e Cura a Carattere Scientifico, Istituto Nazionale dei Tumori, Milan, Italy; Pediatric Neuro-Oncology, Department of Pediatric Oncology, Hospital Sant Joan de Deu, Passeig Sant Joan de Déu 2, Barcelona, Spain; Department of Pediatric and Adolescent Medicine, University Medical Center Mannheim, Medical Faculty Mannheim, Heidelberg University, Mannheim, Germany; Division of Pediatric Hematology and Oncology , University Medical Center Goettingen, Goettingen, Germany; Pediatric Neurosurgery, Charité Universitätsmedizin Berlin, Berlin, Germany; Princess Máxima Center for Pediatric Oncology, Utrecht, The Netherlands; Department of Radiation Oncology, University Medical Center Utrecht, Utrecht, The Netherlands; Division of Pediatric Oncology and Hematology, Department of Women, Child and Adolescent, University Hospital of Geneva, Geneva, Switzerland; CANSEARCH research platform in Pediatric Oncology and Hematology, Faculty of Medicine, Department of Pediatrics, Gynecology and Obstetrics,University of Geneva, Switzerland; Division of Pediatric Hemato-Oncology, Department of Pediatrics and Adolescent Medicine, Medical University of Graz, Graz, Austria; Division of Pediatric Hemato-Oncology, Department of Pediatrics and Adolescent Medicine, Medical University of Graz, Graz, Austria; Princess Máxima Center for Pediatric Oncology, Utrecht, The Netherlands; Department of Neuropathology, Oslo University Hospital, Oslo, Norway; Department of Pathology, Fondazione Policlinico Universitario A. Gemelli IRCCS, Rome, Italy; Institute of Biostatistics and Clinical Research, Faculty of Medicine, University of Münster, Münster, Germany; Sir James Spence Institute of Child Health, Royal Victoria Infirmary, Newcastle upon Tyne, UK; Institute of Neuropathology, Medical Center Bonn, Bonn, Germany; Institute of Neuropathology, Medical Center Bonn, Bonn, Germany; Instituto Estadual Do Cérebro Paulo Niemeyer and the IDOR Institute, Rio de Janeiro, Brazil; Division of Pediatric Hematology and Oncology , University Medical Center Goettingen, Goettingen, Germany

**Keywords:** diffuse midline glioma, DIPG, pediatric high-grade glioma, World Health Organization

## Abstract

**Background:**

The WHO Classification of Tumors of the Central Nervous System has undergone major restructuring. Molecularly defined diagnostic criteria were introduced in 2016 (revised 4th edition) and expanded in 2021 (5th edition) to incorporate further essential diagnostic molecular parameters. We investigated potential differences between specialists in perception of these molecularly defined subtypes for pediatric high-grade gliomas (pedHGG).

**Methods:**

We designed a 22-question survey studying the impact of the revised 4th edition of the WHO classification on pedHGG. Data were collected and statistically analyzed to examine the spectrum of viewpoints and possible differences between neuro-oncologists and neuropathologists.

**Results:**

465 participants from 53 countries were included; 187 pediatric neuro-oncologists (40%), 160 neuropathologists (34%), and 118 additional experts (26%). Neuro-oncologists reported issues with the introduction of molecularly defined tumor types, as well as the abolishment or renaming of established tumor entities, while neuropathologists did not to the same extent. Both groups indicated less relevant or insufficient diagnostic definitions were available in 2016. Reported issues were classified and assessed in the 2021 WHO classification and a substantial improvement was perceived. However, issues of high clinical relevance remain to be addressed, including the definition of clinical phenotypes for diffuse intrinsic pontine glioma and gliomatosis cerebri.

**Conclusions:**

Within the WHO classification of pediatric brain tumors, such as pedHGG, rapid changes in molecular characterization have been introduced. This study highlights the ongoing need for cross talk between pathologist and oncologist to advance the classification of pedHGG subtypes and ensure biological relevance and clinical impact.

**Key Points**

- Perspectives differ on molecular diagnoses between oncologist and pathologist in pediatric HGG.
- The 2021 WHO CNS classification is a substantial improvement with many issues addressed.
- Definitions for clinical phenotypes like DIPG still need addressed.

**Importance of the Study**

Feedback from the greater neuro-oncology community on the introduction of molecular diagnoses into pediatric high-grade glioma (pedHGG) is missing. We bring into focus the clinical and tissue-based diagnostic issues by comparing the perceptions and experiences of pediatric neuro-oncologists and neuropathologists, providing a representative overview of needs within pedHGG management and the WHO classification. Furthermore, we assess and discuss if and how issues raised within the survey have been addressed in the 2021 WHO Classification of Tumors of the Central Nervous System (CNS). Our study underlines the ongoing need to balance advances in the understanding of the biology of CNS tumors with meaningful clinical impact, but also reassures the substantial improvement for definition and diagnostics of pedHGG within the latest WHO classification.

The 5th edition of the WHO Classification of Tumors of the Central Nervous System (CNS5) is now available,^1^ and its summary has been published.^2^ The new edition further increases the role of molecular diagnostics for some CNS tumor types, initialized in the revised 4th edition (CNS4).^3^ For pediatric high-grade glioma (pedHGG) in particular, major changes were implemented following advances in the understanding of genomic and epigenomic landscapes, including the discovery of histone H3 mutations.^4,5^ In 2016, based on several biopsy studies, the diagnosis of diffuse intrinsic pontine glioma (DIPG), a primarily neuroradiological characterized entity until that point, was molecularly defined as diffuse midline glioma (DMG), H3K27M-mutant. In 2021, this tumor type was expanded to DMG H3K27-altered,^2,3^ such that H3K27-wildtype DMGs display (like H3K27M-mutant DMGs), loss of H3K27 trimethylation, but carry other underlying molecular events than K27M mutations.^6,7^ Such rapid reclassification and fundamental changes in nomenclature have resulted in debates between clinicians and pathologists with regard to the implementation of the WHO classification and its impact on diagnostics and treatment of pedHGG patients in daily routine.

The CNS5 (2021) is a substantial refinement of the revised CNS4 (2016). It was generated over the last three years after extensive evaluation of the current status by an expert panel, “cIMPACT-NOW” (Consortium to Inform Molecular and Practical Approaches to CNS Tumor Taxonomy – Not Officially WHO).^8–14^ General feedback from the greater neuro-oncology community on the introduction of molecular diagnoses however is still missing. We therefore conducted a worldwide survey among specialists involved in the diagnosis and therapy of pediatric brain tumors. The survey was created by largely focusing on the CNS4-related issues that were brought up during meetings of the European Society for Paediatric Oncology High Grade Glioma Working Group (SIOPE HGG WG) following the publication of CNS4. The main issues identified by the SIOPE HGG WG were: the introduction of molecularly vs clinically defined DMG and the issue that other pedHGG tumor (sub)types had not been adequately addressed. Furthermore, issues were raised by the SIOPE HGG WG about access to technology and socioeconomic factors involved in molecular diagnostics in pedHGG. These issues have already been covered separately.^15^ Here, we bring into focus the clinical and tissue-based diagnostic issues by a comparison of the different perceptions and experiences of pediatric neuro-oncologists and neuropathologists on this subject, providing a representative overview for the specific needs with regard to pedHGG management and WHO classification. Since CNS5 was published in the meantime with further major changes for pedHGG tumor subtypes, we were able to assess and discuss if and how the various issues raised with our survey have been addressed in this update.

## Methods

The survey was designed and pretested by the European Society for Paediatric Oncology High Grade Glioma Working Group (SIOPE HGG WG). An online version of the survey was created using SurveyMonkey® (San Mateo, Ca, USA). Addressees of this survey study were primarily neuro-pathologists, pediatric neuro-oncologists, neurosurgeons, radiation oncologists, neuroradiologists, and other professionals in the field of pediatric neuro-oncology between March 22 and May 8, 2019. These professionals were identified using contact lists from a prior international survey within the International Society of Neuropathology (ISN),^16^ from the SIOPE Brain Tumour Group, the German Society of Pediatric Oncology and Hematology (GPOH), the German Neuro-oncology Working Group (NOA), the German Society of Neuropathology and Neuroanatomy (DGNN), as well as other international collaborators in the field of pediatric neuro-oncology. Multiple replies from the same IP and/or email address were excluded.

The survey consisted of twenty-two questions, twelve “Yes” or “No” questions, eight multiple choice questions, and two demographic questions. Within each thematic section we identified one key question. Respondents who failed to answer four out of six predefined key questions (including questions 1, 3, 10, 14, 16, and 17) were excluded. All key questions were dichotomous, “Yes or No”. Key questions covered subjects including (1) awareness of the revised 2016 WHO classification, (2) awareness of the newly introduced entity diffuse midline glioma (DMG), H3K27M mutant, (3) opinions on the upcoming 5th WHO classification regarding introducing infantile glioma, (4) introducing pediatric subtypes for anaplastic astrocytoma and glioblastoma, (5) introducing anaplastic pilocytic astrocytoma grade III and, (6) removing gliomatosis cerebri (Supplementary Appendix A). Inclusion and exclusion criteria of respondents for survey analysis were consistent with methods used in Baugh et al.^15^ Data were analysed using Pearson’s Chi-square and Fisher’s Exact Test in IBM SPSS Statistics version 26 (Armonk, NY, USA). Research involving human subjects according to the World Medical Association Declaration of Helsinki did not apply, thus ethics approval was not required for this study. Independent professionals, no patients, were asked for voluntary participation. No personal identifying data were collected and participation did not involve any advantage, disadvantage, or any potential harm.

## Results

The survey was completed by 482 participants, of whom 17 (4%) were excluded for not completing the predefined minimum key questions as outlined above. Participants included 187 pediatric neuro-oncologists (40%), 160 neuropathologists (34%), and 118 (26%) other specialists in the field. The latter group included 45 neuroradiologists (10%), 29 radiation oncologists (6%), 20 neurosurgeons (4%), eight adult neuro-oncologists (2%), seven scientists (2%), and nine nonspecified specialists (2%). Geographically, most participants were from Europe (*n* = 291; 62%), followed by North America (*n* = 59; 13%), Asia (*n* = 49; 11%), Latin America (*n* = 36; 8%), Oceania (*n* = 11; 2%), and Africa (*n* = 8; 2%), 11 (2%) respondents could not be geographically allocated (Table 1). In total, 53 different countries were represented in the survey.

**Table 1.** Survey Participants by Specialization and Location

Specialty No. (%)	Africa	Asia	Europe	Latin America	North America	Oceania	Not Specified	Total
Neuro-oncologists	3	12	121	12	27	6	6	**187** (40%)
Neuropathologists	4	21	91	16	25	3	0	**160** (34%)
Other^a^	1	16	79	8	7	2	5	**118** (26%)
**Total**	**8** (2%)	**49** (11%)	**291** (62%)	**36** (8%)	**59** (13%)	**11** (2%)	**11** (2%)	**465**

^a^ Neurosurgeons, radiation oncologist, neuroradiologists, adult neurooncologists, scientists, and not specified.

**Figure 1.**
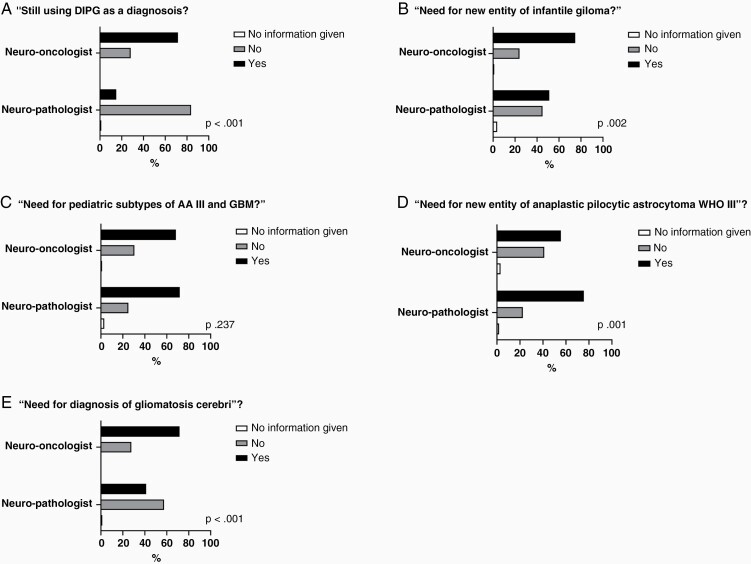
Participant feedback on key questions regarding the revised CNS4.

Key issues from five specific pedHGG areas from the CNS4, defined by the SIOPE HGG WG, were surveyed: (1) DMG/DIPG, (2) infantile glioma (referred to as “infant-type hemispheric glioma” in CNS5), (3) specific (diffuse) pedHGG subtypes, (4) anaplastic pilocytic astrocytoma WHO grade III, and (5) gliomatosis cerebri. Results from questions representing these key areas are displayed in Figure 1. Participating pediatric neuro-oncologists more often prefer using the diagnosis of DIPG than neuropathologists, i.e., 72% vs 15% respectively (survey question 5, *P* < .001; Figure 1a). When further asked why and when one would still use the term DIPG, most oncologists stated using both terms, DIPG and DMG, depending on context (survey question 6, answer b) while, interestingly, the majority of pathologists still agreed that “Diffuse midline glioma, H3K27M mutant, does not cover all DIPG” (survey question 6, answer d).

On the need for introduction of infantile (high-grade) glioma as a new tumor entity, 75% of pediatric neuro-oncologists were in support, in comparison to 51% of neuropathologists (survey question 10, *P* .002; Figure 1b). Argumentation for introducing infantile glioma varied, as neuropathologists indicated that “genetic findings including methylation suggest a tumor entity of its own” (survey question 11, answer b), whereas oncologists argued that “therapy is usually different from high-grade gliomas of older children and adults” (survey question 11, answer c). The term “infantile hemispheric glioma” corresponds to a DNA methylation class. The corresponding tumor type was finally named “infant-type hemispheric glioma” in the WHO CNS5.

Concerning specific pediatric high-grade glioma subtypes (distinct from adult high-grade glioma) and the presumed need to introduce a subtype for anaplastic astrocytoma and glioblastoma in children (3 years and older), both specialties were in support, 68% and 72% of oncologists and pathologists, respectively (survey question 14, *P* .237; Figure 1c). Agreement was also found on the reasoning, with a majority from each group selecting “genetic findings including methylation suggest specific pediatric subtypes of anaplastic astrocytomas/glioblastomas” (survey question 15, answer b). However, regarding adding a new tumor type for “anaplastic pilocytic astrocytoma, WHO grade III”, pathologists were more in favor with 76% in support, in comparison to 56% oncologists (survey question 16, *P* .001; Figure 1d).

On the topic of a diagnosis for gliomatosis cerebri, neuro-oncologists were more in support than neuropathologists, i.e., 72% vs 41% respectively (survey question 17, *P* < .001; Figure 1e). The majority in support of the diagnosis from both groups selected their reasoning as, “diagnosis for a specific phenotype of an underlying glioma, but not as a tumor subtype or entity of its own” (survey question 18, answer a).

**Figure 2.**
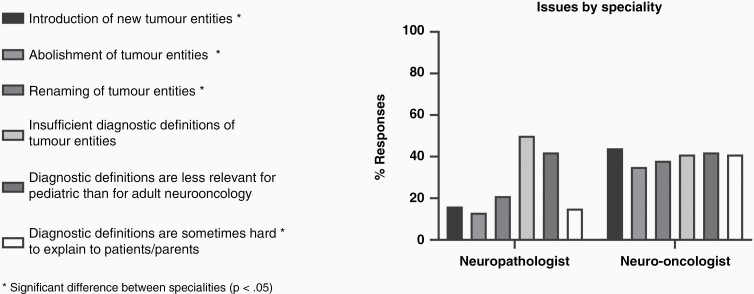
Participant feedback on issues with the revised CNS4.

Finally, overall experiences with the revised 4th edition were collected from 57% of all participants, who reported having issues with the classification. The specific issues surveyed are displayed in Figure 2. Neuro-oncologists significantly more often stated that “the introduction of new tumor entities” caused issues, 44% vs 16% of neuropathologists (survey question 20, answer a; *P* < .001), followed by difficulty with “the abolishment of tumor entities”, 35% vs 13% (survey question 20, answer b; *P* < .001), and the “renaming of tumor entities”, 38% vs 21% (survey question 20, answer c; *P* .004). Neuro-oncologists also reported that “diagnostic definitions are sometimes hard to explain to patients/parents”, 41% vs 15% (survey question 20, answer f; *P* < .001). Feedback was not significantly different on the topics including; “insufficient diagnostic definitions of tumor entities”, 50% of pathologist in support and oncologists 41% (survey question 20, answer d; *P* .20), and lastly, for “diagnostic definitions are less relevant for pediatric than for adult neuro-oncology”, 42% for both groups (survey question 20, answer e; *P* .90) (Figure 2).

## Discussion

Five editions of the WHO CNS Tumor Classification are now available, with the first edition published in 1979.^17^ Later editions followed in 1993, 2000, 2007, 2016, and 2021.^1,18–20^ The pace of discoveries in recent decades has greatly improved our understanding of pediatric brain tumor pathogenesis. This has led to the invention, reinvention, and fine-tuning of a classification system that is now largely based on molecular genetics. The 2016 revised 4th edition was the first large scale molecular restructuring of the WHO CNS Tumor Classification, with the introduction of an “integrated approach” utilizing both pheno- and geno-type.^3^ This new approach affected diffuse glioma as well as embryonal tumors. This system was devised from the ISN-Haarlem Consensus Guidelines in 2014. During development, particular focus was placed on balancing molecular advances with practical issues arising from molecular classifications being incorporated into patient management and diagnosis.^21^

Our present study, underlines the ongoing need to balance molecular advances with meaningful clinical impact in pedHGG. Here, we compared the respective perspectives of the two key players at both ends of this balance, i.e. the neuropathologists as representatives for the focus on the scientific state of the art diagnostics, and the pediatric neuro-oncologists with their special focus on clinical needs. Among the participating specialists, particularly neuro-oncologists reported having issues with the introduction of new tumor types, renaming, or abolishment of established tumor types, while neuropathologists did not. Neuro-oncologists also cited diagnostic definitions being difficult to explain to patients and families. Neuro-oncologists and neuropathologists however agreed on the points that insufficient diagnostic definitions were available for molecular-based entities in 2016 and that these entities were less relevant for pediatric cases (Figure 2).

Interestingly, many of the issues raised in our survey are mirrored by the changes made in the 2021 CNS5. In 2016 CNS4, some arguably clinically relevant pedHGG tumor types like nondiffuse pilocytic astrocytoma, IDH-wildtype diffuse pedHGG, and diffuse pedHGG in infants younger than 3 years of age were not included, but are now specifically addressed (Table 2). “Entities” not included in the CNS5, DIPG and gliomatosis cerebri, are both imaging-defined. In our survey, generally more pathologists accepted the removal of the designation “gliomatosis cerebri” than oncologists. This was also the case with DIPG. Neuro-oncologists were in favor of re-establishing the option of the previous clinical radiological diagnosis of DIPG, in addition to the sole option of setting the DMG diagnosis by biopsy only. It should be noted that in the CNS5, DIPG is listed in a new section entitled “related terminology”, as an acceptable definition.

**Table 2.** Comparisons Between Participant Feedback on the Revised CNS4 in 2016 and Changes Implemented in the CNS5 in 2021

Paediatric HGG WHO 2016	Relevant Survey Questions Addressing the Issue	Problem Confirmed By Survey Results	Addressed by WHO 2021?	Pediatric HGG WHO 2021
1. Diffuse midline glioma, H3K27M mutant 2. DIPG removed as neuroradiological diagnosis	Neuroradiologically defined DIPG diagnosis still needed?	Yes: 46.9%	No	No change
		No: 52.7%		
	H3 wildtype DIPG with poor prognosis as own subtype needed?	Yes: 73.3%	Yes	Two subtypes of DMG, H3 wildtype with loss of H3K27 trimethylation defined: 1. DMG, EZHIP overexpressed 2. DMG, EGFR mutant
		No: 21.5%		
Anaplastic astrocytoma, IDH wildtype and Glioblastoma, IDH wildtype	Pediatric subtypes of anaplastic astrocytoma and glioblastoma needed?	Yes: 68.6%	Yes	Two new entities of pediatric diffuse high- grade glioma: 1. Diffuse pediatric high-grade glioma, IDH/ H3 wildtype 2.Diffuse hemispheric glioma, H3.3G34 mutant
		No: 29.5%		
	New entity “infantile glioma” for high-grade gliomas in infants < 3 years needed?	Yes: 61.7%	Yes	Infant-type hemispheric glioma as new entity of diffuse high-grade glioma in infants
		No: 35.9%		
Pilocytic astrocytoma with anaplastic features analogous to WHO III	“Anaplastic pilocytic astrocytoma WHO III” needed?	Yes: 63.4%	Yes	Pilocytic astrocytoma with anaplasia is still present. The new entity “high grade astrocytoma with piloid features” does not represent the pediatric anaplastic pilocytic astrocytoma
		No: 32.3%		
Gliomatosis cerebri removed as a neuroradiological diagnosis	Neuroradiological defined diagnosis of gliomatosis cerebri still needed?	Yes: 58.7%	No	No change
		No: 40.0%		

For DIPG/DMG, there remains no curative treatment approach with radiation as the palliative therapeutic mainstay. Prognostic differences within DMG subtypes have emerged, with H3.1 K27M-mutant tumors conferring a relative survival advantage over H3.3 K27M-mutant and H3K27-wildtype tumors.^22^ However, outcomes remain universally poor with an 11-month median overall survival.^23^ Tumor subtyping requires a biopsy to be performed in specialized centers, and preferably in the context of clinical trials, given targeted therapies are purely investigative at this point.^24^ Moreover, imaging exams are also generally more available to clinicians than to pathologists, forming a routine part of their clinical decision making. Oncologists will at some point find themselves in the situation where a treatment decision needs to be made, and if no definite molecular-based diagnosis could be rendered, at least an imperfect surrogate (i.e. imaging) can support decision making.

Why imaging defined tumor types like DIPG are not incorporated in the CNS5 is based on the decision that the WHO classification follows a tissue-based approach. When molecular analysis could not (or not successfully) be performed and therefore diagnosis is histology-based only, the classification system advises to add the term “NOS” (not otherwise specified). Imperfect surrogates to molecular classification are required particularly in the context of no biopsy and/or when advanced molecular analyses are not possible. In such a situation for DIPG, a limited immunohistochemistry (IHC) stain for mutant H3 K27M protein or loss of H3 K27 trimethylation can be performed. IHC demonstration of loss of H3 K27 trimethylation may also enable detection of the newly introduced CNS5 DMG diagnoses, with wildtype H3 K27 and absent H3 K27 trimethylation associated with EZHIP protein overexpression and/or EGFR alterations.^6^ IHC staining for H3 K27 trimethylation and H3 K27M appears sufficiently indicative in comparison to molecular sequencing, beyond it is cost-effective and efficient.^25^

For less advanced national health systems where molecular analyses may not be available, the clinical radiological diagnosis of DIPG, as performed for more than 20 years, represents an affordable and clinically meaningful surrogate test for the diagnosis of pontine DMG.^15^ This consideration is supported by a lack of effective therapies available, based on the presence of H3 K27M mutation. And, when there are H3 K27M-specific therapies in future, clinical radiological diagnosis of DIPG would still include most, if not all H3 K27M mutant DIPG.^26^ Furthermore, it remains unclear if all DIPG diagnosed by clinical radiological criteria are indeed sufficiently covered by the CNS5 diagnoses of DMG. According to von Bueren et al., up to 15% of DIPGs display H3 K27 wildtype, with a similarly poor prognosis as H3.3 K27M mutant DIPG.^27^ By now, it remains speculative if these 15% of DIPG are all characterized by loss of H3K27 trimethylation and really fitting into the present range of DMG, H3K27-altered. If neuroradiologically defined DIPG with a similarly poor prognosis of DMG are indeed not fully covered by CNS5, then the consideration of introducing an additional neuroradiological layer for WHO CNS Tumor Classification might be helpful in future.^26^

The tension between clinical relevance and keeping pace with advances in science and technology has been evident in the development of prior versions of the WHO CNS Tumor Classifications. The WHO grade I-IV system for CNS tumors for example was controversial at the time of development. Derived in the concept of “clinical malignancy”, it sought to associate meaningful clinical prognosis, with histologic parameters. This numeric grading was seen as imperfect and of limited utility by some contributors, yet in practice verbal grading was already being carried out, necessitating a formalized grading system.^28^ The challenge today to correlate molecular findings with meaningful clinical significance is much the same. It is well demonstrated that genotype and epigenetics are of clinical significance in pediatric high-grade glioma, but should not eliminate clinical phenotyping, as both provide relevant complementary information. For example, meaningful new predictors in the future could include information about immune status or tumor microenvironment, when single cell sequencing or liquid biopsies are more commonly performed.

Future research will surely help discern whether clinical correlates with biology result in improved therapeutic response and outcome and inform new iterations of the WHO CNS Tumor Classification. Increased multidisciplinary representation within working groups such as the cIMPACT-NOW, with more neuro-oncologists, neuroradiologists, and others involved in the treatment of brain tumor patients could help improve clinical translation. Importantly, representation from countries with a limited access to molecular diagnostics can help inform adaptation of the WHO CNS Tumor Classification to resource-limited settings. Furthermore, inclusion of patients from sites in middle and low income countries will be required to enable robust and powered clinical trials utilizing stratification by pediatric tumor subtype.^29^ Without inclusion of these patients into large international trials, there is a concern that clinical studies will be hindered by too small biological groups.^30^ The challenge remains to improve molecular diagnostic capabilities within low resourced settings and in turn improve the applicability of the WHO classification for CNS tumors.

## Conclusions

In the quest to classify pediatric high-grade gliomas utilizing the most up to date research, the WHO CNS classification has made substantive improvements in incorporating molecular information into the diagnosis of several tumor types. Our study underlines the ongoing need to balance advances in the understanding of the biology of CNS tumors with meaningful clinical impact, but also reassures the substantial improvement for definition and diagnostics of pedHGG within the latest WHO classification. Many points of criticism in the revised CNS4 have been addressed in CNS5. Nevertheless, upcoming WHO CNS Tumor Classifications should continuously work towards improved molecular stratification with a meaningful emphasis on clinical pathological correlation in a multidisciplinary fashion.

## Supplementary Material

vdac077_suppl_Supplementary_Appendix_S1
