# Supplementary material for: Pediatric high-grade gliomas and the WHO CNS Tumor Classification—Perspectives of pediatric neuro-oncologists and neuropathologists in light of recent updates
Source: Neurooncol Adv. 2022 May 20;4(1):vdac077. doi: 10.1093/noajnl/vdac077 (PMC9209749; doi:10.1093/noajnl/vdac077)
Supplement: vdac077_suppl_Supplementary_Appendix_S1 [file vdac077_suppl_supplementary_appendix_s1.docx]

**Appendix A.** Full Length Survey

**Pediatric HGG and YOUR experience with the revised WHO classification**

1. Are you aware of the revision of the WHO Classification of Tumours of the Central Nervous System that

occurred in 2016?

*If you are a neuropathologist who needs to work with the revised classification please don't feel offended and*

*continue :) ...*

- No
- Yes

2. Do you use the revised WHO Classification in your daily practice?

- No
- Yes

3. Are you aware of the newly introduced tumour entity “diffuse midline glioma, H3K27M mutant (WHO grade

IV)”?

- No
- Yes

4. Do you use the diagnosis of diffuse midline glioma, H3K27M mutant?

- No
- Yes

5. Do you still prefer DIPG (“diffuse intrinsic pontine glioma”) as neuroradiological/clinical diagnosis instead of

diffuse midline glioma, H3K27M mutant, when located within the pons?

- No
- Yes

6. **If you answered YES to the previous question** (*"Do you still prefer DIPG as neuroradiological/clinical*

*diagnosis instead of diffuse midline glioma ...?"*), please specify why or when you still use the term DIPG

(several answers are possible):

- DIPG is a well defined and established diagnosis/diagnostic term
- I use both terms depending on the respective context
- Patients can better understand DIPG as diagnosis than diffuse midline glioma, H3K27M mutant
- Diffuse midline glioma, H3K27M mutant, does not cover all DIPG
- Any other answer?_____________

7. Do you believe there is an entity of DIPG, **H3K27 WILDTYPE**, WHO IV?

- No
- Yes

8. How would you treat a child (3 years and older) with a diffuse astrocytoma WHO grade II of the pons,

**H3K27 WILDTYPE**, which fulfils clinical/neuroradiological criteria of DIPG?

- Like a low grade glioma
- Like a diffuse midline glioma, H3K27M mutant
- If using different protocols for diffuse midline gliomas, H3K27M mutant, and other high grade gliomas: Like other high grade
- gliomas
- Individually, depending on other genetic findings including methylation

9. Do you think there is a need to introduce a new tumour entity of **“Diffuse midline glioma of the pons,**

**H3K27 WILDTYPE (WHO grade IV)**” with typical neuroradiological features of a DIPG?

- No
- Yes

10. Do you think there is a need to introduce a new tumour entity of “infantile glioma” for histologically

diagnosed high grade gliomas in infants younger than 3 years?

No

Yes

11. **If you answered YES to the previous question** (*"Do you think there is a need to introduce a new tumour*

*entity of “infantile glioma” ..."*), please specify why (several answers are possible):

- Prognosis is usually significantly better
- Genetic findings including methylation suggest a tumour entity of its own
- Therapy is usually different from high grade gliomas of older children and adults
- Any other reason? _____________

12. If you think that there is indeed a need for a new tumour entity of “infantile glioma” would you classify this

new entity as

- WHO grade I
- WHO grade II
- WHO grade III/IV (depending on histological grade like it is now)
- Individually depending on genetic findings including methylation signature
- Without a defined WHO grade

13. What do you think about routine analysis of IDH status in paediatric anaplastic astrocytomas and

glioblastomas?

- Not adequate because of low percentage (<10%) of IDH mutant paediatric HGG
- Obligatory for all cases
- Only if sufficient tumor material is available
- I don´t know
- Any other comment? _____________

14. Do you think there is a need to introduce new “paediatric subtypes” for anaplastic astrocytomas and

glioblastomas in children (3 years and older) and adolescents/young adults?

- No
- Yes

15. **If you answered YES to the previous question** (*"Do you think there is a need to introduce new*

*“paediatric subtypes” for anaplastic astrocytomas and glioblastomas ..."*), please specify why (several answers

are possible):

- Prognosis is usually better than in older adults
- Genetic findings including methylation suggest specific paediatric subtypes of anaplastic astrocytomas/glioblastomas
- Any other reason? _____________

16. Do you think there is a need to introduce a new tumour entity of “Anaplastic pilocytic astrocytoma (WHO

grade III)” or “Anaplastic astrocytoma with piloid features (WHO grade III)”, respectively, for pilocytic

astrocytomas with anaplastic features?

- No
- Yes

17. Do you think there is still a need for diagnosis of gliomatosis cerebri with typical neuroradiologcal features

of diffuse growth pattern involving two and more cerebral lobes ?

- No
- Yes

18. **If you answered YES to the previous question** (*"Do you think there is still a need for diagnosis of*

*gliomatosis cerebri ..."*), please specify (several answers are possible):

- Diagnosis in the meaning of a SPECIFIC PHENOTYPE of an underlying glioma, but not as a tumour subtype or entity of its own
- Diagnosis in the meaning of a SPECIFIC TUMOUR SUBTYPE of its own for an underlying glioma histology
- Diagnosis in the meaning of a TUMOUR ENTITY of its own independently of an underlying glioma histology
- Any other suggestions? _____________

19. In summary, has the implementation of the revised WHO Classification caused any problems?

- No
- Yes

20. **If you answered YES to the previous question** (*"In summary, has the implementation of the revised*

*WHO Classification caused any problems?"*), please specify your relevant issues (several answers are

possible):

- Introduction of new tumour entities
- Abolishment of tumour entities
- Renaming of tumour entities
- Insufficient diagnostic definitions of tumour entities
- Diagnostic definitions are less relevant for pediatric than for adult neurooncology
- Diagnostic definitions are sometimes hard to explain to patients/parents
- Any other problems? _____________

21. What is your field of expertise?

- Paediatric Oncologist/Paediatric Neurooncologist
- Neuropathologist
- Neurosurgeon
- Radiotherapist
- Radiologist/Neuroradiologist
- Scientist/Biologist/Physician Scientist
- Any other field of expertise? _____________

22. In which country are you working?

and________________________________ YOUR experience
